# Investigation on the Machinability of Polycrystalline ZnS by Micro-Laser-Assisted Diamond Cutting

**DOI:** 10.3390/mi15101275

**Published:** 2024-10-21

**Authors:** Haoqi Luo, Xue Wang, Lin Qin, Hongxin Zhao, Deqing Zhu, Shanyi Ma, Jianguo Zhang, Junfeng Xiao

**Affiliations:** 1 State Key Laboratory of Intelligent Manufacturing Equipment and Technology, School of Mechanical Science and Engineering, Huazhong University of Science and Technology, Wuhan 430074, China; m202370528@hust.edu.cn (H.L.);; 2 Beijing Precision Engineering Institute for Aircraft Industry, Aviation Industry Corporation of China, Beijing 100076, China; 3 Shanghai Aerospace Control Technology Institute, Shanghai 201109, China

**Keywords:** polycrystalline ZnS, in-process-heat laser-assisted diamond machining, brittle-to-ductile transition, soft brittle material

## Abstract

Polycrystalline ZnS is a typical infrared optical material. It is widely used in advanced optical systems due to its excellent optical properties. The machining accuracy of polycrystalline ZnS optical elements must satisfy the requirements of high-performance system development. However, the soft and brittle nature of the material poses a challenge for high-quality and efficient machining. In recent years, in situ laser-assisted diamond cutting has been proven to be an effective method for ultra-precision cutting of brittle materials. In this study, the mechanism of in situ laser-assisted cutting on ultra-precision cutting machinability enhancement of ZnS was investigated. Firstly, the physical properties of ZnS were characterized by high-temperature nanoindentation experiments. The result revealed an increase in ductile machinability of ZnS due to plastic deformation and a decrease in microhardness and Young’s modulus at high temperatures. It provided a fundamental theory for the ductile–brittle transition of ZnS. Subsequently, a series of diamond-cutting experiments were carried out to study the removal mechanism of ZnS during in situ laser-assisted cutting. It was found that the mass damage initiation depth groove generated by in situ laser-assisted cutting increased by 57.99% compared to the groove generated by ordinary cutting. It was found that micron-sized pits were suppressed under in situ laser-assisted cutting. The main damage form of HIP-ZnS was changed from flake spalling and pits to radial cleavage cracks. Additionally, the laser can suppress the removal mode difference of different grain crystallographic and ensure the ductile region processing. Finally, planning cutting experiments were carried out to verify that a smooth and uniform surface with Sa of 3.607 nm was achieved at a laser power of 20 W, which was 73.58% better than normal cutting. The main components of roughness were grain boundary steps and submicron pit. This study provides a promising method for ultra-precision cutting of ZnS.

## 1. Introduction

Zinc sulfide (ZnS) is an important infrared optical material with excellent optical transmittance and mechanical and thermal properties. Hot isostatically pressed ZnS (HIP-ZnS) is also known as multispectral ZnS due to its high transmittance in the band of 400 nm–1200 nm. HIP-ZnS has great application prospects in infrared optics and aerospace. At present, it is widely used in infrared imagers, measuring instruments, fairing and focusing lenses for laser systems, etc. For infrared optical elements, the lower the surface roughness and number of surface defects, the smaller the total scattering value of the workpiece [1,2] and the greater the optical properties of the elements. Additionally, surface and subsurface damage layers reduce the laser-damaged threshold and induce component failure. HIP-ZnS is a typical soft–brittle material [3,4], and conventional grinding could induce great subsurface damage. Diamond-cutting technology is an ultra-precision machining method with the capability of processing high-precision and low-damage surfaces. However, it is prone to brittle fracture during the ordinary cutting process due to the low fracture toughness of HIP-ZnS. As a superior infrared material [5], how to improve the machining quality and efficiency of ZnS has become a critical challenge.

Extensive research has been conducted on the cutting mechanisms of polycrystalline ZnS, particularly regarding its behavior in brittle and ductile regimes. Li et al. [6] integrated the material properties of ZnS with the machining mechanics of hard and brittle materials, investigating its turning characteristics via micro-morphological analysis and XRD spectra. Their findings indicated that the likelihood of ductile cutting increases when the crystal axis is aligned with the cutting direction. Zhang et al. [7] proposed a modified stress-assisted nanocutting model based on the virtual boundary between the workpiece and tool, exploring the nanocutting process under various external stresses. Arif et al. [8] introduced a cutting energy-related model to predict the brittle-to-ductile transition in diamond turning of polycrystalline brittle materials, accounting for workpiece properties, tool geometry, and cutting parameters. Yang et al. [9] developed an ultra-precision diamond turning process with trapezoidal modulation, maintaining constant chip thickness and cutting direction, offering new strategies to enhance the brittle-to-ductile transition and machining performance. Similarly, Yao et al. [10] calculated energy consumption in both ductile and brittle cutting modes during ultra-precision machining of ZnS, establishing the critical uncut chip thickness for brittle-to-ductile transition and outlining the necessary machining conditions. However, despite these advancements, surfaces produced through the diamond turning of ZnS still exhibit crater-like defects due to particle fragmentation, limiting the precision fabrication of optical components.

To better understand the ZnS damage mechanisms during machining and address the associated challenges, extensive research has been conducted. Salzman et al. [11] utilized magnetorheological finishing with chemically modified fluids to investigate the material-removal behavior of single-crystal ZnS, aiming to address the challenges posed by its anisotropy. While this technique proved effective, its lower machining efficiency compared to turning and limitations in processing optical components with complex geometries—such as aspherical, freeform, and diffractive surfaces—present challenges. In contrast, Guo et al. [12] explored the influence of cutting parameters on ZnS surface quality through an orthogonal experimental approach, optimizing parameters to achieve a surface roughness of 2.3 nm. Zong et al. [13] proposed an innovative oblique-cutting model to enhance surface quality by facilitating a brittle–ductile transition during machining. The effectiveness of this method was verified through turning experiments and finite element simulations, offering new insights into the plastic machining of ZnS. Building on this, Navare et al. [14] employed micro-laser-assisted single-point diamond turning to study the effects of diamond tool crystal orientation on machining outcomes, presenting new strategies for improving ZnS surface quality. Despite these advances, the microscopic deformation mechanisms of polycrystalline ZnS during cutting remain insufficiently explored. Chen et al. [15] categorized the brittle fracture of ceramic matrix composites into macroscopic and macroscopic scales based on distinct material units. Zheng et al. [2] developed a coarse-grained molecular dynamics approach to systematically investigate the effects of material properties and cutting depth on removal patterns. Their transitional depth of cut (TDoC) model accurately predicted the material removal behavior, offering a robust framework for analyzing the cutting dynamics of polycrystalline ZnS. Unlike single-crystal materials, polycrystalline materials experience non-uniform fracture during machining due to the effects of crystal orientation, grain boundaries, twins, and grain size. Consequently, optimizing diamond-cutting technology is crucial to mitigate these non-uniform fractures and enhance the machinability of polycrystalline materials.

Lasers are widely used in the ultra-precision machining of various materials as an assistance due to their high energy density, easy control, and small heat-affected zone. In situ laser-assisted machining improves the ductile cutting properties of materials by focusing a laser beam through the diamond tool on the cutting zone. In situ laser-assisted machining has been applied in the manufacturing of brittle materials in recent years. It has been demonstrated that silicon [16], germanium [17], calcium fluoride [18], zinc selenide [5], tungsten [19,20], tungsten carbide [21], silicon carbide [22], microcrystalline glass [23], and fused silica [24] can be processed with smooth surface by in situ laser-assisted cutting. He et al. [25] carried out molecular dynamics simulations of Ti alloy laser-assisted machining. They discussed the effect of laser power on the micro-deformation and damage of the material and verified the laser-assisted suppression of the subsurface damage layer. Ma et al. [26] investigated the effect of different machining parameters on the surface roughness of alumina ceramics. They characterized the surface morphology after processing and found that the crystal breakage was compacted to form a dense layer heated by a laser. Cracks were significantly inhibited, and the integrity of the processed alumina ceramic surface was improved. Pu et al. [27] characterized and compared the 3D morphology of laser-assisted cutting Si_3_N_4_ surfaces under different material removal modes and concluded that the relationship between different material removal modes and 3D morphology parameters. Surface roughness decreases with increasing laser power. Kong et al. [28] analyzed and compared the differences in chip morphology of titanium alloys in OC and LAC. They revealed that chips from in situ laser-assisted cutting are excessive from the continuous type to the segmented type, with less stress in the shear region and faster formation of shear bands. Yang et al. [29,30,31] first investigated the deformation mechanism, nano-wear mechanism, and material removal mechanism of KDP crystals using the molecular dynamics method. They established scratching maps to minimize the subsurface damage of KDP crystals. They carried out simulations of nano-scratches at different depths under different crystal orientations. It was found that the anisotropy of the material has a great influence on the deformation mechanism of KDPs under nano-wear. Based on this, yang established scratching maps as a guideline for quantitatively defining the “depth/radius removal mechanism” on different lattice surfaces of KDPs, which contributed to the study of the failure mechanism of KDPs. This contributes to the study of the failure mechanism of KDPs. Liu et al. [32] investigated the influence of temperature on the removal mechanism of materials by nano-scratch experiment. They revealed that the hardness and elastic modulus of the material change with temperature and explained the high ductility of KDP crystals at high temperatures, which provides a basis for the low-damage machining of soft and brittle materials under a thermal field. Geng et al. [5] verified the feasibility of laser-assisted processing techniques for improving the processing efficiency and surface integrity of ZnSe ceramics through comparative and orthogonal experiments. Unlike common single-crystal hard brittle materials (silicon, germanium, silicon carbide, etc.), ZnS is a typical polycrystalline soft brittle material. With lower hardness and fracture toughness, it is susceptible to fracture during diamond cutting, reducing surface quality. ZnS is more similar to ZnSe, but ZnS has higher fracture strength, hardness, and flexural strength. The grain size of ZnS is about 150 μm, which is larger than ZnSe with a grain size range of 30–50 μm.

In summary, although some scholars have studied the cutting mechanism and process of ZnS, the problems of low efficiency and low surface quality of ZnS cutting in machining have not been solved. In situ lasers can improve the material properties of the cutting area, reducing surface and subsurface damage and facilitating the enhancement of ductile machinability of soft and brittle materials. However, the mechanism of in situ laser-assisted cutting of ZnS has not been investigated. This study explores the main damage forms and material removal mechanisms of HIP-ZnS crystals in in situ laser-assisted cutting. Firstly, high-temperature nanoindentation studies were carried out to reveal the influence of temperature on the hardness, elastic modulus, and plastic deformation capacity of ZnS. Subsequently, in situ laser-assisted cutting experiments on HIP-ZnS were carried out. Optical microscopy and a white light interferometer (WLI) were utilized to characterize and analyze the material removal mode and main damage forms during the cutting process. The results revealed that the property differences of different grains were suppressed to a certain extent with laser irradiation, and, thus, the material cutting performance and machined surface quality were improved. The large critical undeformed chip thickness can be applied to the HIP-ZnS turning process, which is conducive to the improvement of the material removal rate. It provides a promising method for ultra-precision manufacturing of HIP-ZnS.

## 2. Materials and Methods

### 2.1. Materials

**Figure 1:**
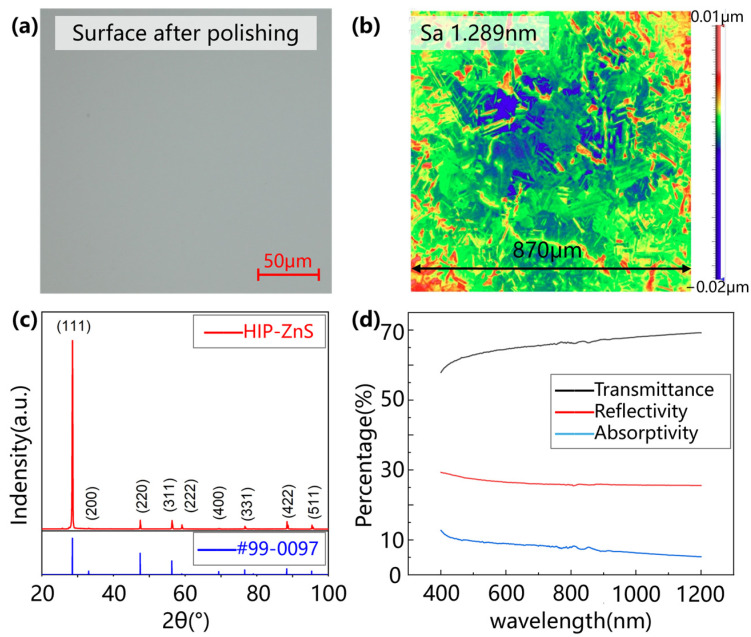
Characterization of ZnS samples. (**a**) Polished surface by optical microscope; (**b**) polished surface by WLI; (**c**) XRD pattern; (**d**) absorption spectra of the used workpiece.

In this study, multispectral ZnS crystals were used, which were supplied by the GRINM Guojing Advanced Materials Co., Ltd. (Beijing, China). The multispectral ZnS crystals were prepared by applying hot isostatic pressure treatment (HIP) to standard ZnS crystals made by chemical vapor deposition (CVD). HIP-ZnS is polycrystalline, and the grains in HIP-ZnS are mainly present as face-centered cubic crystals (FCC). The FCC ZnS undergoes cleavage in the form of a dodecahedron with six cleavage directions and a perfect cleavage surface of (011). Twin crystals are mostly present in aggregates parallel to <111> and <211>. A white light interferometer (Newview 9000, Zygo Corporation, Middlefield, CT, USA) was used to observe the polished surface quality of the samples. The polished surface of the multispectral ZnS crystals is shown in Figure 1a,b, with a surface roughness Sa (arithmetic mean height of the plane) of less than 2 nm. The phase composition of the material was characterized and analyzed by X-ray diffraction (XRD 7000, Shimadzu corporation, Kyoto, Japan). As shown in Figure 1c, the diffraction intensity of the sample is mainly from α-phase ZnS (sphalerite crystal type). The grains in the multispectral HIP-ZnS crystals are all in the α-phase, and <111> is their major crystallographic orientation. The absorbance, transmittance, and reflectance of the material were tested by a UV–visible near-infrared spectrophotometer (SolidSpec-3700, Shimadzu corporation, Kyoto, Japan), and, as shown in Figure 1d, the absorbance of the HIP-ZnS crystals for the light of 1064 nm wavelength was 5.9015%.

### 2.2. High-Temperature Nanoindentation

**Figure 2:**
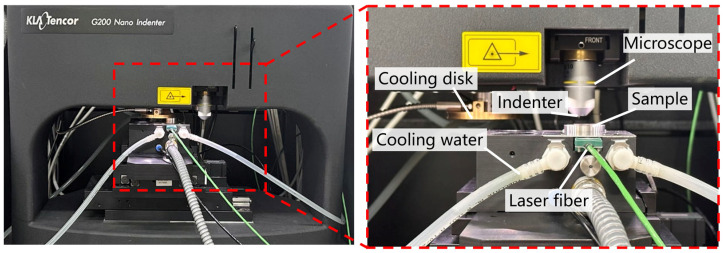
High-temperature nanoindentation equipment.

The high-temperature nanoindentation experiment generates the load-displacement curve of the material to obtain the mechanical properties such as microhardness and Young’s modulus of the material. The plastic deformation capacity of the material can be known by analyzing the curve. The maximum undeformed chip thickness of the material is correlated with the microhardness, Young’s modulus, and fracture toughness of the material. Conducting high-temperature nanoindentation experiments can provide theoretical support for ZnS groove experiments. High-temperature nanoindentation experiments were carried out on polished ZnS samples (15 mm × 15 mm × 1 mm) using a high-temperature nanoindenter (Nano Indenter^®^ G200, KLA foundation, Ann Arbor, MI, USA), as shown in Figure 2. The tip radius of the diamond Berkovich probe was about 100 nm, and the fusion angle was 142.30°. The diamond indenter is susceptible to oxidation at high temperatures, and the thermal drift of the indenter will increase dramatically. This leads to a large shift in the displacement-load curve and a decrease in the reliability of the experimental results. The laser heating temperature of the experimental platform and the limited maximum platform temperature determined that the ambient temperature used in the nanoindentation test was 25 °C. High-temperature conditions are set at 100 °C, 200 °C, 300 °C, and 400 °C. In this study, five nanoindentations were performed at five temperature conditions to collect load depth data in order to remove random error effects and ensure more credible results. The nanoindentation interval was set to 350 μm based on the grain size and grain distribution of the ZnS crystals. The triboindenter was operated in the load-controlled mode, with constant loading and unloading rates for every test cycle. The maximum load was 20 mN, the loading and unloading times were 10 s, and the load holding time was 2 s. A Bruker in situ SPM imaging system was used to obtain the indentation morphology. Prior to the indentation experiment, the sample stage was heated by a laser beam with the heating and cooling rate set to 10 °C/min, which heated the sample to a set temperature using the principle of heat conduction. Subsequently, the probe tip was moved into the heated test chamber to ensure isothermal contact conditions between the indenter and the sample. We automatically adjusted the thermal drift of the probe within 3 nm/s using the program to achieve thermal equilibrium between the sample and the probe.

### 2.3. Grooving Experiments

**Figure 3:**
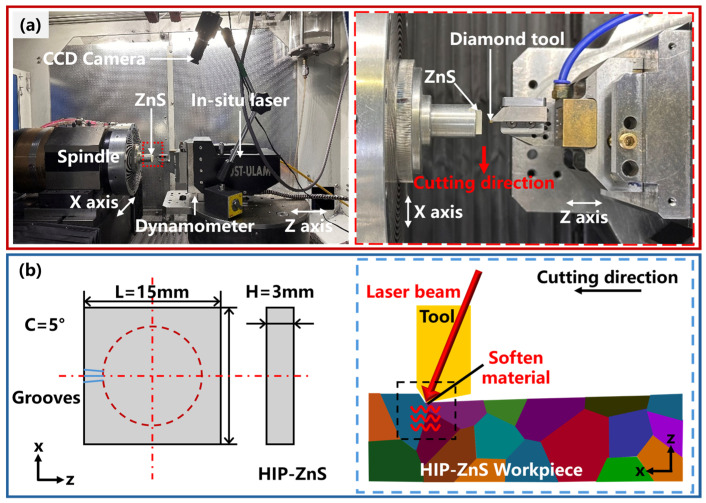
Cutting experiment: (**a**) equipment and (**b**) machining schematic diagram.

The experiment was conducted on an ultra-precision machine tool (Nanoform X, Precitech, Keene, NH, USA) with a self-developed in-LADM system, as shown in Figure 3a. Groove experiments and planning experiments were performed to study the machinability improvement of HIP-ZnS materials with laser assistance, as shown in Figure 3b. Figure 3b shows the schematic diagram of groove experimental machining, and the experiment was carried out on a four-axis machine tool. Multiple grooves are machined by rotating the spindle. C represents the angle of each groove spacing and, in order to reduce the influence of different grain directions on the experiment and reduce the systematic error of the experiment, the value of C is taken to be 5°. L represents the length dimension of the workpiece used, and H represents the thickness dimension of the workpiece. The workpiece used in this experiment is a 15 mm × 15 mm × 3 mm rectangular sample. The laser generator utilizes Nd:YAG as the active medium, providing the continuous-wave laser with a wavelength of 1064 nm, which has a Gaussian profile. The laser beam was guided through a specially designed optical beam shaper module, focusing the laser beam from 3 mm to about 80 μm in diameter. Laser applied to near-transparent diamond tool edges, heating the workpiece by adjusting the laser power in the range of 0–25 W. Based on the simulation results of COMSOL software version 6.1, the material temperature at the center of the laser spot reaches 1200 °C when the laser power comes to 30 W. The center temperature is higher than the sublimation temperature of ZnS, and the material is prone to high-temperature oxidation and ablation. A power of 25 W was selected as the highest laser power. Since the laser beam exhibits a Gaussian distribution, there was a large temperature gradient around the laser irradiation area given by the short groove cutting distance and large feed rate. To attenuate its effect, the laser spot position is precisely adjusted to coincide with the center of the diamond tip. No cutting fluid was used in the groove-cutting experiments to minimize thermal stress. We used a multispectral absorbing power meter (30 (150) A-BB-18, Ophir, Jerusalem, Israel) to ascertain that the cutting interface received the proper amount of laser power and that the laser emitted through the right region of the tool. A single-crystal diamond (SCD) tool was used to feed horizontally at a constant cutting speed along the X direction, while the depth of cut was continuously varied along the Z direction from 0 to 4 μm during the groove cutting process. The SCD tool used in the experiment had a tip radius of 0.479 mm, a rake angle of −35°, and a clearance angle of 10°. The shift between the removal modes of HIP-ZnS crystals under OC and LAC was analyzed by groove experiments. The detailed machining conditions are shown in Table 1. Furthermore, planning experiments were carried out to verify the effect of laser on the machinability of the material during the continuous cutting process. The detailed machining conditions are shown in Table 2.

### 2.4. Surface Characterization

The morphology of the grooves was observed by an optical microscope (Carl Zeiss Axiolab 5, Oberkochen, Germany), and the main forms of defects in the material and the influence of twinning and grain orientation were analyzed. After the planning experiments, a white light interferometer (WLI, Newview 9000, Zygo Corporation, Middlefield, CT, USA) was used to observe the cutting planes to verify the effect of material grain orientation, grain boundaries, and twins on the material removal mode.

## 3. Results and Discussions

### 3.1. Temperature Response of Material Properties

**Figure 4:**
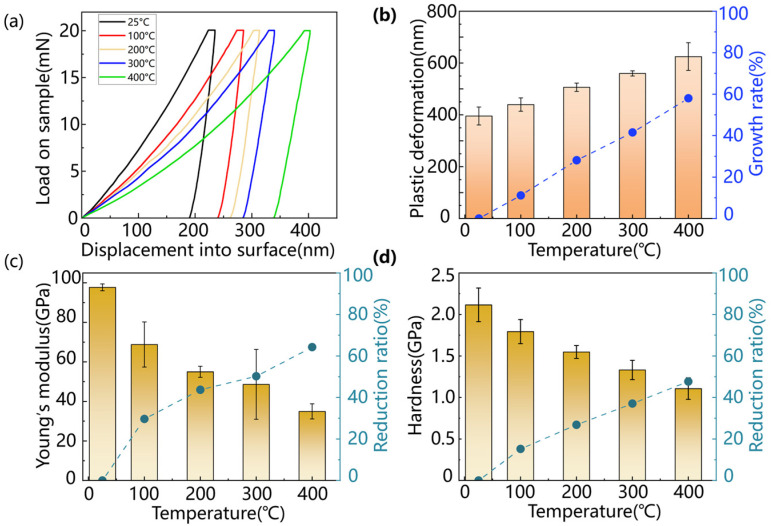
High-temperature nanoindentation results: (**a**) load–displacement curves under different temperatures; variation in (**b**) plastic deformation, (**c**) Young’s modulus, and (**d**) materials’ hardness with increasing temperatures.

Figure 4a depicts the load-displacement curves of the material at temperatures of 25 °C, 100 °C, 200 °C, 300 °C, and 400 °C. The results show that the load-displacement curves changed significantly with increasing temperature and the indentation became deeper under the same load condition. The average Young’s modulus of HIP-ZnS was 97.73 GPa at room temperature and 55.01 GPa and 34.95 GPa at 200 °C and 400 °C, respectively, as shown in Figure 4c. The average microhardness of ZnS was 2.12 GPa at room temperature and 1.55 GPa and 1.11 GPa, as shown in Figure 4d. Both Young’s modulus and hardness of HIP-ZnS decreased significantly with increasing temperature: Young’s modulus decreased by 64.25% and hardness decreased by 47.69%. Dislocation activity is enhanced at high temperatures and the efficiency of cleavage increases with temperature. By transferring energy to the material by the laser, the defects in the crystal are more likely to be displaced, making the material more susceptible to plastic deformation. The stiffness of the material decreases during the softening process at high temperatures, which makes it easy to produce large elastic deformation, and its ductile deformation ability is enhanced, as shown in Figure 4b, and the depth of ductile deformation increases from 395.53287 nm at 25 °C to 624.8939 nm at 400 °C, which is conducive to obtaining a good surface quality and a desirable tool life.

### 3.2. Material Cutting Removal Mechanism

**Figure 5:**
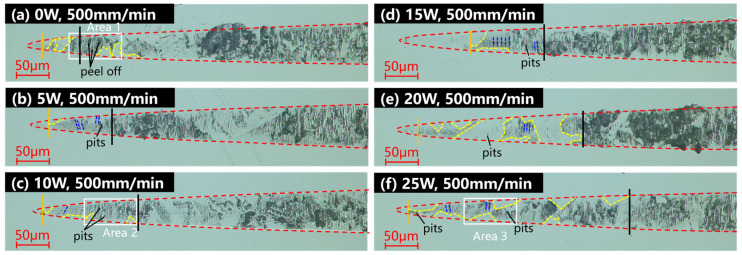
Power comparison grooving experimental results under (**a**) laser power of 0 W, cutting speed of 500 mm/min, (**b**) laser power of 5 W, cutting speed of 500 mm/min, (**c**) laser power of 10 W, cutting speed of 500 mm/min, (**d**) laser power of 0 W, cutting speed of 500 mm/min, (**e**) laser power of 5 W, cutting speed of 500 mm/min, (**f**) laser power of 10 W, cutting speed of 500 mm/min.

Grooving experiments were carried out to investigate the influence of in situ lasers on the machinability of HIP-ZnS. Investigate the changing pattern of the material-removal mechanism with the change of the in situ laser power and cutting speed. Firstly, the grooving experiments were performed at laser powers of 0 W, 5 W, 10 W, 15 W, 20 W, and 25 W, and the experimental results are shown in Figure 5. The area comprising the yellow dashed line in the groove is the ductile region, and the dark blue dashed line is demarcated as the cleavage cracks. When grooving under OC at a small depth of cut, the material is removed in ductile form, and a smooth surface is presented in the groove, as shown in Figure 5a. As the depth of cutting increases, the material first undergoes violent brittle fracture, forming large crater defects on the surface. Subsequently, a large crack is presented in the groove, and the percentage of ductile removal of the material remains at a high level. When the depth of the cut reaches around 160 nm, the crack density increases, and the material is removed in a brittle mode. The onset depth of extensive chipping is defined as the depth at which the percentage of damage in the groove exceeds 60%, as shown by the black line in Figure 5. Accompanying the increase in power, the black lines in Figure 5a–f gradually move towards the back end of the grooves, indicating that the depth of large-area chipping initiation is increased. The depth of ZnS initiation of the large-area brittle fracture is increased in the presence of a laser, which improves the depth of the cut in planning and is beneficial for obtaining high-quality ZnS surfaces with high effectiveness. Under 25 W laser power, the material is removed in ductile form at small depths of cut. As the depth of cut increases, the cutting force increases, the material undergoes cleavage, and the cracks appear in the grooves, during which the crack density and crack size increase and gradually evolve into craters, as shown in Figure 5f. By analyzing the machined grooves, it was found that most of the material at the front of the grooves was removed plastically in the grooves under different experimental conditions. However, brittle fractures still occurred in some grains, producing craters and cleavage cracks. Compared with the material removal process in OC, the localized damage stage of grooves in LAC has a larger area of ductile area and reduced cratering and spalling. Cleavage cracks are produced in polycrystalline materials due to the occurrence of cleavage during mechanical processing. They are a common brittle processing defect in polycrystalline materials. Compared with the point-like and flake-like craters produced by grain crushing and pulling out, the cleavage cracks are regarded as brittle fractures of a lower degree. It is also seen that, with the reduction in crater defects in the grooves with increasing power in the grooves under different experimental conditions, the brittle defects are more in the form of cleavage cracks, which are considered to be an improvement in processing properties. Thus, the laser energy field can improve the material cutting properties and enhance the machining quality. In the interest of further investigation of the effect of thermal density on the material, a set of rate comparison tests at the same laser power is carried out.

**Figure 6:**
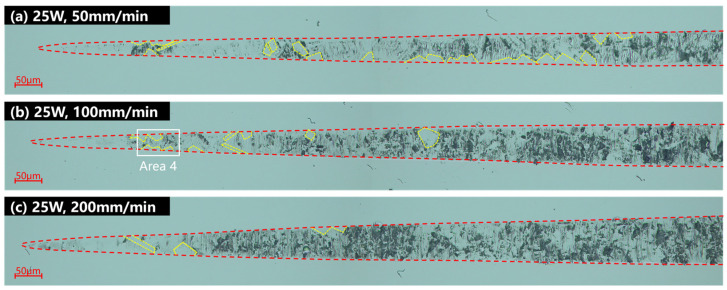
Cutting speed comparison grooving experimental results under (**a**) laser power of 25 W, cutting speed of 50 mm/min, (**b**) laser power of 25 W, cutting speed of 100 mm/min, (**c**) laser power of 25 W, cutting speed of 200 mm/min.

Under the laser power of 25 W, the grooving experiments were carried out at cutting speeds of 200 mm/min, 100 mm/min, and 50 mm/min, respectively, and the experimental results are shown in Figure 6. The area bounded by the yellow dashed line in the groove is the ductile region, and the dark blue dashed line labels the cleavage cracks. The onset of the brittle–ductile transition phase is marked with an orange solid line, and the onset of large-scale collapse is marked with a black solid line. Compared with the experimental results of the 500 mm/min cutting speed shown in Figure 5f, the area of the ductile removal region at the initial of the groove increases with the increasing cutting speed, but there are still a few defects. This is due to localized brittle chipping caused by individual grains being pulled out due to their hardness. The location where cracks and pits commence in the grooves to the point where large-scale collapse initiates is defined as the brittle–ductile mixing removal stage, as indicated by the orange line in Figure 5 and Figure 6. In the ductile–brittle mixing removal stage, the density and size of the craters in the grooves decrease with the reduction of the cutting speed. This is consistent with the reduced microhardness, Young’s modulus, and ductile deformability of the material at high temperatures. The increase in laser energy density improves the cutting performance of HIP-ZnS.

**Figure 7:**
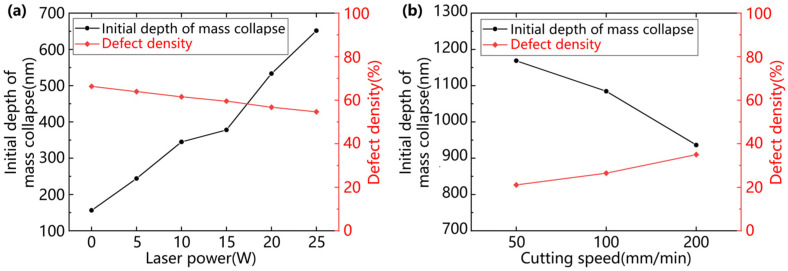
Initial depth of mass collapse chipping and damage density curves: (**a**) power comparison experiment; (**b**) cutting speed comparison experiment.

Unlike single-crystal materials, the form of material removal at the initial of the grooves under the same processing conditions is influenced by the grain orientation, with the ductile removal region remaining initially locally brittle and the transition region of the brittle–ductile transition being large in size. It was found that, at the beginning of the groove, both brittle and ductile removal modes existed. This is due to the different orientations of the grain slip systems in polycrystalline materials, and the microscopic removal characteristics of the material are closely related to grain orientation and twinning. Although the groove initiation section has a small depth of cut, the cutting force is small, and the cutting energy is not sufficient to sustain crack expansion and consolidation at small depths of cut. However, the hardness of ZnS crystal particles is affected by the crystal direction, and particles with high hardness are pulled out by the cutting force, forming large pits and chipping on the cutting surface. The traditional evaluation index of cutting performance is the brittle–ductile transition depth, which is difficult to use to accurately describe the changes in the processing performance of the material. As the depth of cut increases further, the cutting force increases, more cracks are generated and undergo expansion and merging, and the crack density increases rapidly and is eventually distributed over the entire groove surface. At this point, the material is removed in a brittle mode, so the depth of large chipping initiation is selected as the cutting performance evaluation index in this study. The onset depth of extensive chipping is defined in this study as the depth at which the percentage of damage in the groove exceeds when 60%, as shown by the black line in Figure 5. ‘Damage’ is defined as defects such as craters, cleavage cracks, etc., formed by brittle fracture. It is reflected in the optical microscope picture of the groove as a dark part with a different color from the polished surface. In this paper, an image recognition method was used to write a program to identify the density of defects in the grooves and to calculate the percentage of damage in the grooves. At the same time, in order to quantitatively study the changes in the brittle–ductile mixing region under the laser-assisted action and to reduce the influence of special individual cases, the defect density in the groove in the interval of 150 nm–300 nm was selected as an auxiliary evaluation index. As shown in Figure 7, the onset depth of large-area chipping of ZnS grooves increases with the increase in laser power, and the defect density of grooves in the 150 nm–300 nm interval decreases with the increase in laser power.

**Figure 8:**
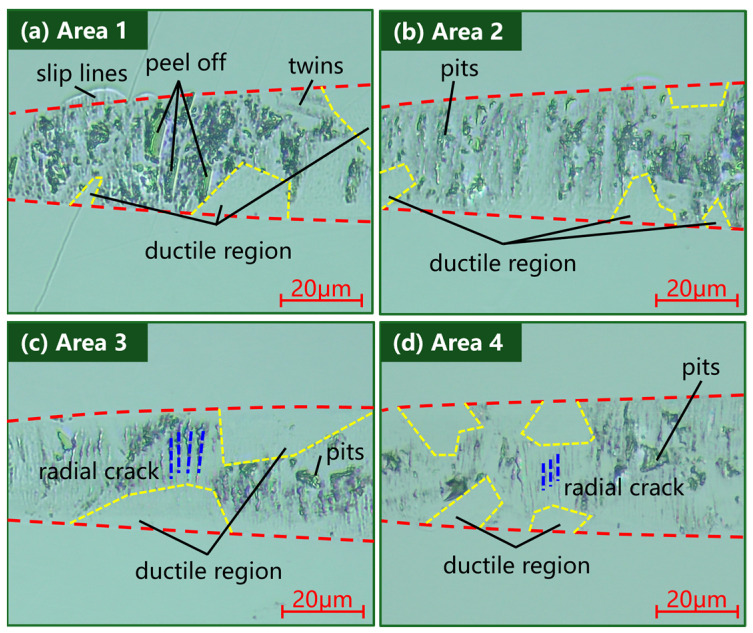
Results of the grooving experiment: (**a**) laser power of 0 W, cutting speed of 500 mm/min; (**b**) laser power of 10 W, cutting speed of 500 mm/min; (**c**) laser power of 25 W, cutting speed of 500 mm/min; (**d**) laser power of 25 W, cutting speed of 100 mm/min.

In pursuit of investigating whether the laser can inhibit the cutting variability of different grain orientations and sizes, the grooves were analyzed by using an optical microscope with higher magnification to analyze the grooves. The grooves in the 150 nm–300 nm interval under the experimental conditions of (a) laser power of 0 W, cutting speed of 500 mm/min, (b) laser power of 10 W, cutting speed of 500 mm/min, (c) laser power of 25 W, cutting speed of 500 mm/min, and (d) laser power of 25 W, cutting speed of 100 mm/min were selected as the objects of analysis, as illustrated by the white areas in Figure 5 and Figure 6. As shown in Figure 8a, in the absence of a laser, large grains undergo cleavage, forming pits and flaky flakes on the material surface. Twinned crystals undergo cleavage, producing discrete diagonal banding patterns on the surface. As shown in Figure 8b, under the action of a 10 W laser, the material softens at a high temperature, the hardness decreases, the fracture toughness increases and the crater formed by the fracture becomes smaller. The material undergoes cleavage along the direction perpendicular to the cutting direction, and the cleavage cracks are mostly craters and flaky spalling. As shown in Figure 8c, in 25 W laser-assisted cutting, the ductile domain area in the grooves increased, and the main form of damage to the material changed from pits to radial cleavage cracks. The radial cleavage cracks in the grooves became smaller under the experimental conditions of 25 W–100 mm/min, as shown in Figure 8d. In normal cutting without a laser, the material is removed as a mixture of brittle and ductile in the depth of cut in the interval 150 nm–300 nm. As the hardness of individual grains changes with different grain orientations, the form of removal varies greatly from grain to grain. Currently, the main form of damage to the material is large pits and flake spalling, with a high degree of brittle fracture. In in situ laser-assisted cutting, the material is still removed as a brittle–ductile mix, consistent with normal cutting without the addition of a laser. However, the main form of damage to the material changes. The stiffness of the material decreases at high temperatures, and the grains are more susceptible to transcrystalline fracture rather than intergranular fracture. The material undergoes cleavage mainly in the direction perpendicular to the cutting direction, producing radial cleavage cracks and block spalling in a few grains. As the laser power increased, the craters decreased, the length and width of the cleavage cracks decreased, and the material was removed more in a ductile mode. The reduction of microhardness and Young’s modulus enhanced the ductile processing properties of HIP-ZnS. The high temperature induced by laser irradiation reduces the resistance of the material to local ductile deformation and weakens the grain boundaries, which helps to suppress the effects of crack extension and grain orientation variability on processing. Therefore, a large critical undeformed chip thickness can be applied to the HIP-ZnS turning process with laser assistance, which is conducive to improving the material-removal rate.

**Figure 9:**
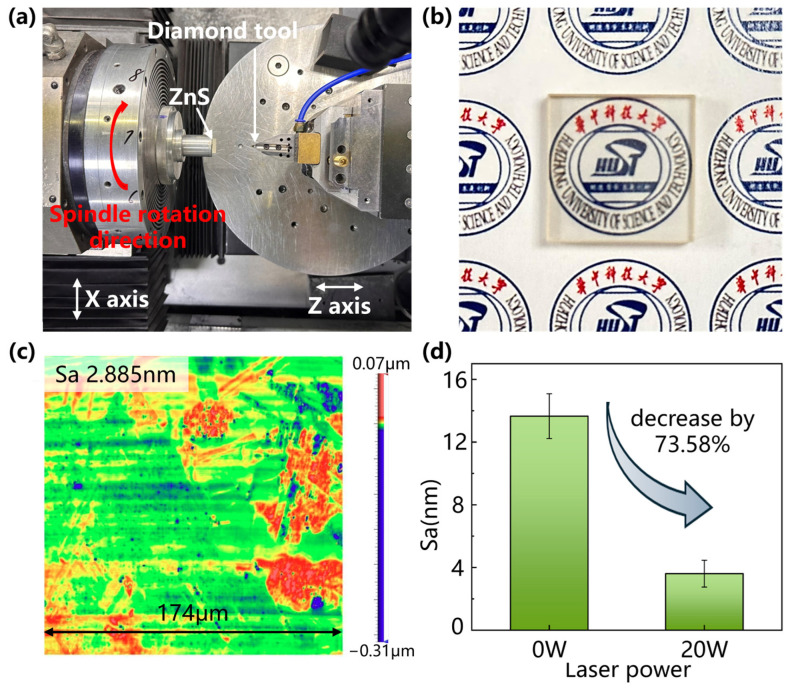
Planning experiments: (**a**) experimental setup; (**b**) processed sample; (**c**) surface quality after experiment; (**d**) surface quality comparison.

To demonstrate laser-assisted enhancement of material machinability, planning experiments of ZnS workpieces were carried out at 0 W and 20 W laser power, respectively. The surface morphology after machining was observed by a white light interferometer in a field of view of 174 μm × 174 μm, as shown in Figure 9. Without the laser, the surface quality was poor, with a roughness Sa of 12.452 nm. The material was removed mainly by crack expansion in brittle mode, with a large number of craters distributed on the surface. In the laser-assisted case, the processed surface quality was better, with a roughness Sa of 2.885 nm. Intermediate cracks, transverse cracks, and pits were suppressed, but a small number of submicron craters and submicron stepped structures were still present on the surface. The reason for this phenomenon is that, in polycrystalline material processing, due to the existence of grain–grain orientation and size variability, ductile material removal of different thicknesses occurs on the crystal boundaries, resulting in the formation of grain boundary steps. The roughness of the tested surface originates from submicron pits and grain boundary steps. The experimental results show that the surface Sa obtained by in situ LADC is reduced by 55.6% compared to conventional single-point diamond turning for surface quality at 20 W laser power, verifying that the ductility of the multispectral ZnS crystals is improved during the in situ LADC process.

**Figure 10:**
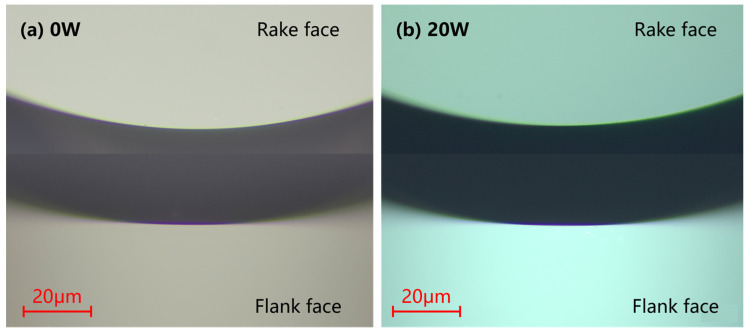
Wear of SCD tool (**a**) under OC and (**b**) under in-LAC.

Figure 10 illustrates a microscopic view of the tool morphology after half an hour of laser-assisted cutting with a distance of 300 m. Figure 10a shows that, due to the low hardness of HIP-ZnS, no significant tool edge fragmentation was observed even for normal cutting without laser. In the picture of the tool after laser-assisted machining shown in Figure 10b, the cutting edge is very sharp, while the front and back cutting surfaces are very smooth. No visible wear bands are seen on the rear tool surface. In situ LADC is a stable cutting process that enables long-term HIP-ZnS machining.

## 4. Conclusions

In this paper, the machinability of ZnS micro-laser-assisted machining was verified. Compared to conventional diamond cutting, the surface quality of ZnS was significantly improved under laser-assisted machining. High-temperature nanoindentation experiments were carried out to investigate the evolution of the crystal properties of polycrystalline HIP-ZnS. The results show that high temperatures can enhance the ductile deformation of HIP-ZnS. The brittle–ductile transition process of the material was investigated by performing OC and in-LAC grooving experiments, and the removal mechanism of the material under in-LAC and OC was investigated using a characterization method. The effects of the thermal field, mechanical properties, and dynamic behavior of the brittle–ductile transition on the surface quality were investigated. Finally, uniform surface quality was experimented by using in situ laser-assisted machining methods. The main conclusions are summarized below:

(1) Thermal effects had a significant impact on the mechanical properties of HIP-ZnS. The depth of ductile deformation of HIP-ZnS increased from 395.53 nm at 25 °C to 624.89 nm at 400 °C, an increase of 57.99%. The microhardness and Young’s modulus decreased with the increase in temperature by 47.69% and 64.25% at 400 °C, respectively. This shows the softening effect of a high temperature on the material and its ability to enhance the ductile deformation of the material.

(2) By OC and in-LAC grooving experiments, it was shown that compared with the conventional cutting, when the laser power was increased to 25 W, the large chipping initiation depth increased from 156.27 nm to 651.74 nm, which is an increase of 318.96%, and the defect density in the interval of 150–300 nm decreased from 66.35% to 54.67%.

(3) The main damage form of HIP-ZnS was changed from lamellar spalling and cratering to small-size cracking under the action of in-LAC. This change is conducive to the formation of surfaces with higher machining quality, indicating that the laser is able to suppress the removal pattern variability of different grain crystallographic directions to a certain extent and ensure the processing of ductile regions.

(4) The results of end face turning verified the superior machining quality of in-LAC over conventional single-point diamond turning. During surface shaping, intermediate cracks, transverse cracks, and pits are suppressed by the additional in situ laser, and the roughness of the measured surface originates from submicron pits and grain boundary steps. At a laser power of 20 W, the surface Sa obtained by in situ LADC was reduced by 73.58%.

In this paper, experiments on ZnS grooving were conducted at different laser powers as well as at different cutting speeds, mainly focusing on the influence of temperature effects on ZnS. The effect of laser assistance on the cutting process of ZnS was explored. To address the shortcomings of this study, the following future work will be proposed: (1) to explore the effect of laser-assisted processing at different wavelengths on the cutting process/removal mechanism of the material; (2) to carry out cutting experiments on ZnS produced by different methods of preparation with different grain sizes or grain orientations; to explore the effect of laser-assisted processing on the cutting process/removal mechanism of ZnS. This study involved ZnS cutting experiments with different grain sizes or grain orientations made by different preparation methods in order to explore the effect of laser-assisted machining and to expand the universality of laser-assisted machining technology to improve the cutting performance of ZnS.

## Figures and Tables

**Table 1:** Conditions of grooving experiments.

Experimental Parameters	Data
Workpiece material	HIP-ZnS
Tool material	Single-crystal diamond
Tool nose radius	0.5 mm
Rake angle	−35°
Clearance angle	10°
Cutting speeds	500 mm/min, 200 mm/min, 100 mm/min, 50 mm/min
Slope ratio	1:1000
Laser power	5 W, 10 W, 15 W, 20 W, 25 W

**Table 2:** Parameters of planning experiments.

Experimental Parameters	Data
Workpiece material	HIP-ZnS
Tool material	Single-crystal diamond
Tool nose radius	0.5 mm
Rake angle	−35°
Clearance angle	10°
Cutting speeds	500 mm/min
Feed rate	4 μm/rev
Depth of cutting	4 μm
Laser power	0 W, 20 W

## Data Availability

The original contributions presented in the study are included in the article, further inquiries can be directed to the corresponding author.
